# CD83 Modulates B Cell Function *In Vitro*: Increased IL-10 and Reduced Ig Secretion by CD83Tg B Cells

**DOI:** 10.1371/journal.pone.0000755

**Published:** 2007-08-15

**Authors:** Birte Kretschmer, Katja Lüthje, Andreas H. Guse, Svenja Ehrlich, Friedrich Koch-Nolte, Friedrich Haag, Bernhard Fleischer, Minka Breloer

**Affiliations:** 1 Department of Immunology, Bernhard Nocht Institute for Tropical Medicine, Hamburg, Germany; 2 Institute of Biochemistry and Molecular Biology I, University Medical Centre Hamburg-Eppendorf, Hamburg, Germany; 3 Institute for Immunology, University Medical Centre Hamburg-Eppendorf, Hamburg, Germany; Centre de Recherche Public-Santé, Luxembourg

## Abstract

The murine transmembrane glycoprotein CD83 is an important regulator for both thymic T cell maturation and peripheral T cell responses. Recently, we reported that CD83 also has a function on B cells: Ubiquitous transgenic (Tg) expression of CD83 interfered with the immunoglobulin (Ig) response to infectious agents and to T cell dependent as well as T cell independent model antigen immunization. Here we compare the function of CD83Tg B cells that overexpress CD83 and CD83 mutant (CD83mu) B cells that display a drastically reduced CD83 expression. Correlating with CD83 expression, the basic as well as the lipopolysaccharide (LPS) induced expression of the activation markers CD86 and MHC-II are significantly increased in CD83Tg B cells and reciprocally decreased in CD83mu B cells. Wild-type B cells rapidly upregulate CD83 within three hours post BCR or TLR engagement by de novo protein synthesis. The forced premature overexpression of CD83 on the CD83Tg B cells results in reduced calcium signaling, reduced Ig secretion and a reciprocally increased IL-10 production upon *in vitro* activation. This altered phenotype is mediated by CD83 expressed on the B cells themselves, since it is observed in the absence of accessory cells. In line with this finding, purified CD83mu B cells displayed a reduced IL-10 production and slightly increased Ig secretion upon LPS stimulation *in vitro*. Taken together, our data strongly suggest that CD83 is expressed by B cells upon activation and contributes to the regulation of B cell function.

## Introduction

CD83 is a type I transmembrane glycoprotein, a member of the Ig superfamily [1], [2] and was initially described as a highly specific marker for activated human dendritic cells (DC) in the peripheral blood [3], [4]. Murine CD83 displays 63% identity to human CD83 in the amino acid sequence and northern blot analysis revealed expression predominantly in the brain and spleen [5] as well as in activated bone marrow derived dendritic cells (BM-DC) [6].

The analysis of mouse strains deficient for CD83 [7], [8] revealed that thymic CD83 expression is crucial for the maturation of double positive thymocytes to single CD4 positive T cells. CD83^−/−^ mice displayed a dramatically reduced amount of single positive T helper cells in the thymus and in the periphery that were restored to normal level by expression of CD83 on thymic epithelial cells. In line with these results the thymic maturation in the presence of ubiquitously expressed soluble CD83Ig fusion protein led to the generation of CD4 positive T cells in normal numbers but with an impaired function [9]. In addition to this central role in the thymic selection of T helper cells there is accumulating evidence suggesting a function for CD83 as an immunological regulator of peripheral T cell responses. Culture in the presence of either a soluble CD83Ig fusion protein or soluble fragments of the extracellular CD83 domain inhibited lymphocyte proliferation in human and murine systems *in vitro* [10]–[12]. Human cytomegalovirus (HCMV) infection induced the shedding of naturally expressed CD83 by infected human DC and this soluble CD83 again suppressed allogenic T cell proliferation [13]. Finally, the administration of recombinant human CD83 inhibited the onset of experimental autoimmune encephalomyelitis (EAE) and cured already induced disease *in vivo* [14].

The mechanism of CD83 mediated immune regulation and the nature of the putative CD83 ligand however, still remain enigmatic [15], [16]. On the one hand human and murine DC upregulated CD83 upon activation [3], [4], [6] and engagement of its putative ligand by CD83 transfected APC increased human T cell activation [17] [18], suggesting that CD83 represents a costimulatory receptor for T cell activation like CD86 and CD80 [19]. On the other hand the expression level of CD83 on DC did not correlate with their capacity to activate murine T cells as shown by three independent studies employing CD83 deficient as well as CD83 overexpressing DC, thus ruling out a non redundant costimulatory function for CD83 on DC at least in the murine system [7], [8], [20].

Monitoring murine CD83 expression pattern and kinetics under conditions of an ongoing *Leishmania major* (*L. major*) or *Trypanosoma cruzi* (*T. cruzi*) infection, we recently demonstrated surface expression of CD83 predominantly on B cells. That CD83 plays an important role on B cells was substantiated by the fact that the ubiquitous transgenic expression of CD83 *in vivo* on the B cells themselves strongly interfered with the production of *L. major* and *T. cruzi* specific Ig as well as with the humoral response to thymus dependent (TD) and thymus independent (TI) model antigens [21].

Here we analyze the impact of CD83 expression on B cell activation *in vitro*. We report that CD83 overexpression induced an increased MHC-II and CD86 expression on CD83Tg B cells whereas diminished CD83 expression on CD83mu B cells resulted in a reciprocally reduced expression of these activation markers. CD83 was rapidly upregulated by activated wild-type B cells within three hours post BCR or TLR engagement. Premature transgenic overexpression of CD83 on B cells at the beginning of stimulation resulted in reduced calcium signaling as well as reduced Ig secretion and reciprocally increased IL-10 production. This phenotype was restricted to the B cell compartment since the CD83Tg T cells displayed normal calcium signaling and proliferation upon *in vitro* stimulation. Furthermore the altered activation of CD83Tg B cells was mediated by CD83 expressed on B cells themselves since it did not depend on the presence of accessory cells. Although reduced CD83 expression did not alter the response of CD83mu spleen cell cultures to LPS stimulation *in vitro*, a reduction in IL-10 release together with a slight increase in Ig production was observed in purified CD83mu B cell cultures. Therefore our data strongly suggest that CD83, expressed on activated B cells, plays a central role in the modulation of B cell function.

## Methods

### Mice and antibodies

All mouse strains employed were bred in the animal facilities of the Bernhard-Nocht-Institute for tropical medicine or in the university hospital Hamburg-Eppendorf (Hamburg, Germany) which are registered by the Federal Health Authorities of the State of Hamburg. Experiments employing mice described within this manuscript are approved by “Amt für Gesundheit und Verbraucherschutz” as 32/05 and 20/07. The animal facilities and the CD83Tg mice founder 2 and founder 1 were generated at the Bernhard-Nocht-Institute [20], Ig HEL BCR transgenic mice (IgHEL Tg) [22] were a kind gift of Prof. Dr. Christian Kurts, (Universität Bonn, Germany). F1 generation of IgHELTg and CD83Tg with C57BL/6 were bred at the Bernhard-Nocht-Institute. CD83 mutant mice (CD83mu, termed LCD4.1 originally) [8] were a kind gift of Prof. Dr. Fred Ramsdell (Zymogenetics, Seattly, WA). Monoclonal antibodies were obtained from BD Pharmingen and Caltag Laboratories. The mAb to mouse CD83 Michel-19 was generated at the Bernhard-Nocht-Institute by immunizing rats with a CD83Ig fusion protein [10]. The polyclonal rabbit antiserum to CD83 was generated at the university hospital Hamburg-Eppendorf by gene gun DNA immunization with murine CD83 full length cDNA.

### Flow cytometry

The Fc receptors of 2×10^5^ spleen cells were blocked with mouse serum (5% v/v) for 10 min on ice. Cells were stained with 1:100 dilutions of the indicated mAb for 20 min on ice and analyzed on a Becton-Dickinson FACS-Calibur equipped with Cell Quest Pro software. Ab and reagents used: FITC-labeled or biotinylated anti-mouse CD83 clone Michel-19; FITC-labeled Rat IgG1, clone R3-34; PE anti-mouse CD19, clone 1D3; PE anti-mouse MHC-II, clone M5/114.15.2; Cy5 affinity purified goat anti-mouse IgM µ-chain specific; PE anti-mouse CD69, clone H1.2F3; PE anti-mouse CD86, clone RMMP-2; PE anti-mouse CD80, clone RMMP-1; FITC anti-mouse CD40, clone 3/23; biotinylated anti-mouse CD138, clone 281-2. Cell death was measured employing the Annexin-V-Fluostaining Kit (Roche, Mannheim, Germany), according to the manufacturer̀s recommendation.

### *In vitro* stimulation of spleen and B cells for CD83 detection

Spleens were prepared from 6 to 10 week old female C57BL/6, CD83Tg founder 1 and founder 2, CD83 negative littermate to CD83Tg founder 1 and CD83mu mice. 2×10^6^ cells were cultured in 2 ml RPMI 1640 medium supplemented with 10% fetal calf serum, 20 mM Hepes and L-glutamine in 24well culture dishes. LPS (10 µg/ml) or anti-BCR (clone 187.1; 1 µg/ml) with or without IL-4 (20 ng/ml) were added. Cells were cultured at 37°C and 5% CO_2_ and triple stained with biotinylated anti-CD83 followed by APC labeled-streptavidin, anti-CD19 FITC and anti-CD69 PE at various time points. Untouched B cells were purified from spleens by magnetic cell sorting employing the Pan B cell isolation kit (Miltenyi Biotec, Bergisch Gladbach, Germany) according to the manufacturer's instructions. Purity of the resulting cell population was analyzed by FACS to be >98% (data not shown). 2×10^6^ purified B cells were incubated with or without 10 µg/ml LPS in 24well culture dishes for 1h and 6h. Cells were harvested and analyzed for CD83 expression by western blot.

### CD83 specific western blot

2×10^6^ B cells were lysed in 50 µl lysis buffer (150 mM NaCl, 50 mM Tris pH 7,4, 1% CHAPS) supplemented with Complete EDTA-free Protease inhibitor (Roche, Mannheim, Germany). For deglycosylation 18 µg protein of each sample was denatured in a total volume of 25 µl with 0,5 µl 10% SDS for 10 min at 70°C. Afterwards 2,5 µl 10% NP40 were added and samples were incubated with 0,5 U N-Glycosidase F overnight. 12 µg protein were loaded in each slot and separated by SDS-Page on a 10–20% PAA gradient gel (Anamed, Darmstadt, Germany) and blotted to an Immobilon-P PVDF membrane (Millipore, Schwalbach, Germany). CD83 was detected by incubating the blocked membrane with a 1∶10.000 fold dilution of the polyclonal rabbit anti-mouse CD83 serum, followed by incubation with a 1∶2000 dilution of HRP conjugated goat anti-rabbit immunoglobulin (Dako, Glostrup, Denmark) and developed with ECL™ Western Blotting Detection Reagents (Amersham Biosciences, Buckinghamshire, England).

### *In vitro* stimulation of spleen and B cells

Whole spleen cells or purified B cells (2×10^5^) derived from C57BL/6, CD83Tg founder 1, CD83 negative littermates to founder 1, CD83Tg founder 2, CD83mu, IgHELTg or IgHEL/CD83 double Tg mice were stimulated with LPS (10 µg/ml) or anti-CD3 (145-2C11, 1 µg/ml) in 0,2 ml RPMI 1640 medium supplemented with 10% fetal calf serum, 20 mM Hepes and L-glutamine in 96 well culture plates. Proliferation was measured by the uptake of ^3^H-thymidine after 48h culture for additional 18h. IL-10 in the supernatant was measured by standard sandwich ELISA employing IL-10 DuoSet ELISA development kit (R&D Systems, Wiesbaden, Germany) according to the manufacturer̀s instructions. Polyclonal Ig was measured in serial dilutions of the culture supernatant after seven days by sandwich ELISA employing 1,25 µg/ml rabbit anti-mouse Ig (polyclonal serum: DAKO #Z0259) as capture and 0.65 µg/ml peroxidase conjugated rabbit anti-mouse Ig (purified fraction of polyclonal antiserum: DAKO # P0260) as detection agent. HEL-specific Ig was measured after seven days culture by ELISA employing HEL (1 µg/ml) as capture antigen and 2 µg/ml biotinylated anti-mouse IgM^a^ (clone DS1) as detection mAb. To determine HEL-specific Ig *in vivo*, blood was obtained from six to ten week old, naïve IgHELTg and IgHEL/CD83 double Tg mice by puncture of the tail vein. After 60 min coagulation at room temperature serum was harvested by centrifugation and analyzed in serial dilutions for the presence of HEL-specific Ig by ELISA.

### Monitoring of intracellular [Ca^2+^]

Untouched B and T cells were purified from C57BL/6 and CD83Tg mice by magnetic cell sorting employing Pan-B cell and Pan-T cells isolation kit (Miltenyi Biotec, Bergisch Gladbach, Germany) and loaded with Fura-2-AM as described [23]. In brief, 3×10^7^ cells were centrifuged at 1500 rpm for 5 min and suspended in 1 ml RPMI 1640 (supplemented with 10% FCS, L-Glutamine; 20 mM Hepes) at 37°C. After an incubation period of 5 min at 37°C, Fura-2 acetoxymethylester (fura-2-AM), the membrane-permeant form of Fura-2, was added to the cells at a final concentration of 4 µg/ml. After a 15 min incubation period, the cell suspension was diluted 5-fold with warm RPMI 1640 and again incubated for 15 min at 37°C. Next the cells were washed and resuspended at a concentration of 6×10^6^ cells per ml in a buffer containing 140 mM NaCl, 5 mM KCl, 1 mM MgSO_4_, 1 mM CaCl_2_, 20 mM HEPES, 1 mM NaH_2_PO_4_, 5.5 mM glucose, pH 7.4. For [Ca^2+^]_i_ monitoring 500 µl cell suspension and 500 µl buffer were transferred into a fluorimeter cuvette. [Ca^2+^]_i_ was monitored at room temperature in a Hitachi F-2000 fluorometer at excitation wavelengths of 340/380 nm (alternating) and an emission wavelength of 495 nm. A stable baseline was generated after approximately 150 s, the stimulant anti-BCR (LO-MK) for purified B cells or anti-CD3 (145-2C11) for purified T cells was added at a concentration of 10 µg/ml and [Ca^2+^]_i_ was monitored for another 10 min. At the end of each measurement the maximal fluorescence (F_max_) was determined by addition of Triton X-100 (10% v/v final concentration) and the minimal fluorescence (F_min_) was determined by addition of EGTA/Tris (400 mM EGTA, 3 M Tris, pH 7.4).

## Results

### CD83 is rapidly upregulated on activated B cells *in vitro*

**Figure 1:**
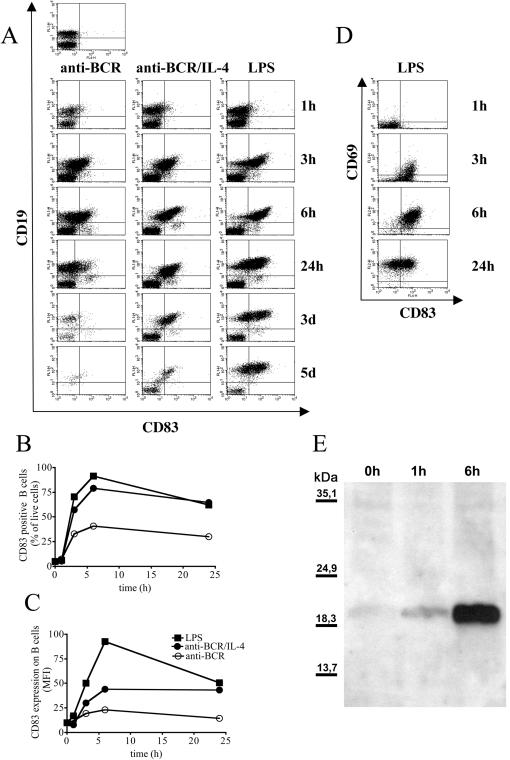
CD83 is upregulated on activated B cells. C57BL/6 mice derived spleen cells (2×10^6^/ml) were stimulated with anti-BCR (1 µg/ml) and IL-4 (20 ng/ml) or with LPS (10 µg/ml) as indicated in the headline. Cells were triple stained for CD19, CD83 and CD69 at the indicated time points. 1A: Dot blots show all lymphocytes positive for surface expression of CD83 on the x-axis and CD19 expression on the y-axis. 1BC: Graphs show the percentage of CD83 positive B cells (1B) or the mean fluorescence intensity (MFI) of CD83 on B cells (1C) after stimulation with anti-BCR alone (open circle) anti-BCR and IL-4 (closed circle) or with LPS (closed square) in an independent experiment, error bars show SEM of duplicates. 1D: Dot blot shows 2×10^4^ CD19 positive cells derived from LPS activated spleen cells analyzed for CD83 (x-axis) and CD69 (y-axis) surface expression. 1E: 2×10^6^ purified C57BL/6 spleen derived B cells were stimulated with LPS (10 µg/ml). B cells were lysed at the indicated time points, deglycosylated and separated by SDS-PAGE. CD83 was detected by western blot with a polyclonal rabbit anti-CD83 serum. Results are representative for at least three independent experiments.

During the course of an ongoing infection with *Leishmania major*, B cells are the dominant CD83 positive cell population and surface expression of CD83 on B cells is strictly correlated with the site and the kinetics of infection [21]. In order to investigate the kinetics of CD83 expression after activation of B cells, we stimulated total spleen cells *in vitro* either with anti-BCR, a combination of anti-BCR and IL-4 or with LPS. Figure 1A shows that surface CD83 expression occurred three hours post stimulation of spleen cells predominantly on CD19 positive B cells. The CD83 staining on activated B cells was successfully competed by pre-incubation with unlabeled anti-CD83 mAb or with a CD83Ig fusion protein but not with CD28Ig fusion protein (data not shown). CD83 expression on B cells reached a maximum at six hours post stimulation in cultures stimulated via BCR- or TLR-engagement. While both stimuli induced CD83 upregulation with comparable kinetics, the different nature of the stimuli employed was reflected by differences in the survival of spleen cells at later time points of culture (figure 1A), in the maximum percentage of CD83 positive B cells (figure 1B) and in the intensity of CD83 surface expression (figure 1C). Stimulation with LPS rendered more than 90% of the B cells positive for CD83 six hours after stimulation and induced sustained CD83 surface expression for five days. The engagement of the BCR in the absence of further stimuli however, activated only 30% of the splenic B cells to express CD83 and spleen cells died after two days of *in vitro* culture. Costimulation with IL-4 (figure 1A) or anti-CD40 mAb (data not shown) induced increased and prolonged CD83 expression on B cells as well as prolonged viability of cultures.

In order to analyze if CD83 was specifically expressed on activated B cells in the spleen cell culture, we measured the correlation between CD83 expression and the early activation marker CD69. Figure 1D shows that CD83 expression preceded CD69 expression. While 23% B cells were double positive for CD69 and CD83 at three hours post LPS stimulation, 47% B cells already expressed surface CD83 but still were negative for CD69. CD69 was further upregulated and six hours post LPS stimulation 90% of splenic B cells became double positive for CD83 and CD69. After 24 hours of stimulation, expression of CD83 decreased and CD69 positive CD83 negative B cells occurred for the first time. Stimulation of splenic B cells with anti-BCR and anti-CD40 mAb led to similar results (data not shown).

It has been shown that surface expression of human CD83 upon DC maturation is partially mediated by the transport of intracellular stored CD83 protein to the plasma membrane [24], [25]. To visualize intracellular CD83 in murine B cells, we performed western blots with purified C57BL/6 derived B cells, employing a polyclonal rabbit anti-mouse CD83 antiserum. Hereby CD83 protein migrated in a broad and faint band ranging from 40 kD to 60 kD (data not shown). This was due to different glycosylation of the CD83 protein present in the lysates since deglycosylation changed the CD83 protein migration pattern to a distinct band at approximately 20 kD (figure 1E), which is in line with the predicted molecular weight for a protein consisting of 175 amino acids [5], [6]. Strikingly, no CD83 protein was detectable in lysates of naïve B cell cultures whereas increasing amounts of CD83 protein were present in lysates of B cells that had been LPS activated for one or six hours respectively (figure 1E). This result suggests that activated B cells express CD83 by de novo protein synthesis.

### CD83 expression modulates MHC-II and CD86 surface expression on B cells

In order to investigate a possible impact of activation-induced CD83 on B cell function *in vitro*, we employed mouse strains with manipulated CD83 expression levels. CD83Tg mice (CD83Tg founder 2 and CD83Tg founder 1) overexpress murine CD83 under the control of a MHC-I promoter on every nucleated cell *in vivo* [20]. CD83mu mice (originally named LCD4.1) carry a missense mutation in the stop codon of the CD83 gene leading to a 55 amino acid extension, thus resulting in a severe reduction of CD83 protein expression [8].

**Figure 2:**
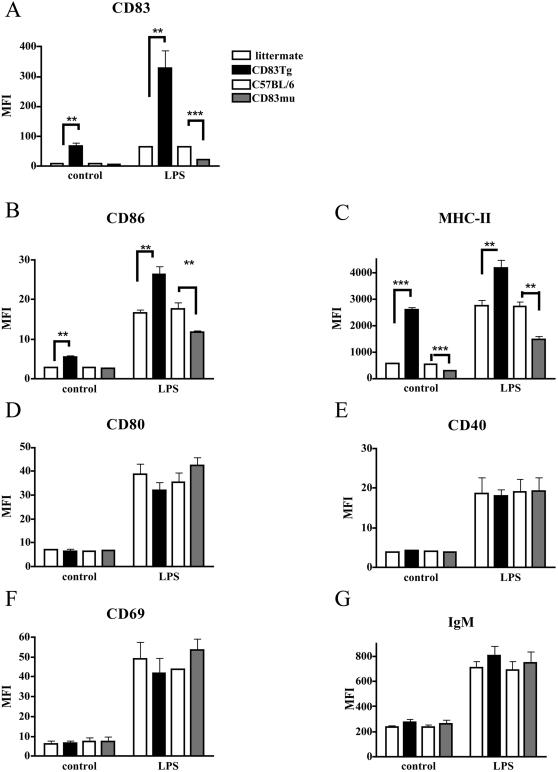
Positive correlation of CD83 expression to CD86 and MHC-II expression on CD83Tg and CD83mu B cells. C57BL/6 and CD83 negative littermates to CD83Tg founder 1 mice (open bars), CD83Tg (black bars) or CD83mu (dark grey bars) derived spleen cells (2×10^6^/ml) were double stained for CD19 and either CD83 (2A), CD86 (2B), MHC-II (2C), CD80 (2D), CD40 (2E), CD69 (2F) or IgM (2G) *ex vivo* or after 48 h incubation with 10 µg/ml LPS as indicated on the x axis. 2×10^4^ CD19 positive cells were analyzed by FACScan. Please note that each bar represents the combined results of five independent experiments employing two female age matched mice of each group per experiment, error bars show SEM. Asterisks indicate a significant difference of the mean (* p<0.05; ** p<0.005; *** p<0.0005) employing students t test.

First we compared expression of different activation markers on B cells derived from wild-type, CD83Tg or CD83mu mice. B cells within whole spleen cell cultures were analyzed for CD83, CD86, CD80, CD69, CD40, MHC-II and surface IgM expression either *ex vivo* or after two days of cultivation in the presence of LPS. As expected, given our previous findings, LPS stimulation induced upregulation of CD83 on wild-type B cells (figure 2A, open bars, either C57BL/6 or CD83 negative littermate to CD83Tg founder 1). CD83Tg B cells displayed an increased CD83 expression on naïve as well as on LPS activated B cells. The observed CD83 upregulation on LPS activated CD83Tg B cells can be due to both endogenous CD83 expression as well as induction of MHC-I promoter controlled transgenic expression of the CD83 transgen. Naïve CD83mu mice in contrast, displayed no detectable CD83 and activation-induced CD83 upregulation was drastically reduced (figure 2A). Interestingly CD83 overexpression led to a significantly increased expression of MHC-II and of the costimulatory receptor CD86 on both naïve and LPS activated B cells while the diminished CD83 expression on CD83mu B cells was correlated to a reciprocally reduced expression of MHC-II and CD86 (figure 2B, 2C). This did not reflect a generalized activated or inhibited phenotype of CD83Tg and CD83mu B cells respectively, since the expression of the early activation marker CD69, the costimulatory receptors CD80 and CD40 as well as surface IgM were normal (figure 2D–2G). Repetition of these experiments with CD83Tg mice derived from an independently generated founder (founder 2) led to similar results (data not shown). Furthermore, this result was reproduced employing purified B cells (data not shown). Taken together these experiments show that CD83 expression is positively correlated to CD86 and MHC-II expression on B cells.

### CD83 overexpression modulates B cell function *in vitro*

**Figure 3:**
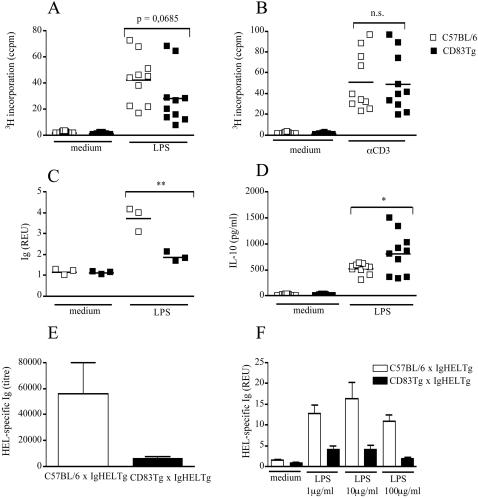
CD83Tg spleen cells display reduced Ig and increased IL-10 response to LPS stimulation. 3A-D: 2×10^5^ spleen cells derived from sex and age matched C57BL/6 (open squares) or CD83Tg founder 2 (closed squares) were cultured in the presence of LPS (10 µg/ml) or anti-CD3 (145-2C11, 1 µg/ml) or no further stimulus, as indicated on the x-axis. 3AB: Proliferation was estimated after 48h by ^3^H thymidine incorporation. 3C: Total mouse Ig in the supernatant was measured after seven days by ELISA. Results are presented as relative ELISA units (REU: OD_450_ sample: OD_450 _medium control) of a 1∶1024 dilution. 3D: IL-10 in the supernatant was quantified by ELISA after 48 h. Each dot represents the analysis of an individual mouse (mean of five replicates the SEM not shown, being below 10%). The lines indicate the median of all mice analyzed and the asterisks indicate a significant difference of the median (*p<0.05; **p<0.005) employing students t test. 3E: HEL-specific Ig in the serum of six IgHEL Tg mice (open bar) or six IgHEL CD83double Tg mice (black bar) was quantified by ELISA, error bars indicate SEM. 3F: 2×10^5^ spleen cells derived from six IgHEL Tg mice (open bars) or from spleen cells derived from IgHEL CD83 double Tg mice (black bars) were cultivated *in vitro* for 48 h in the presence of indicated amounts of LPS. HEL-specific Ig in the supernatant was quantified by ELISA and is presented as REU of a 1: 40 dilution. Error bars show SEM of five replicates. This result is representative for five independent experiments.

To analyze the biological relevance of the correlation between CD83 and MHC-II and CD86 expression, we compared proliferation, Ig and cytokine secretion of CD83Tg, CD83mu and wild-type B cells *in vitro*. First, we assessed the response of CD83Tg (founder 2) and wild-type spleen cells to both, T cell specific and B cell specific stimulation. Proliferation upon LPS stimulation was slightly but non-significantly reduced in CD83Tg spleen cells (figure 3A), while proliferation upon CD3 engagement was comparable thus demonstrating normal T cell function within CD83Tg spleen cell cultures (figure 3B). *In vitro* secretion of LPS induced Ig, however, was significantly reduced in CD83Tg spleen cells compared to C57BL/6 spleen cells (figure 3C) indicating that transgenic expression of CD83 specifically interfered with B cell function *in vitro.* The analysis of cytokines in the culture supernatant revealed increased IL-10 secretion in LPS activated CD83Tg spleen cell cultures compared to wild-type spleen cell cultures (figure 3D), arguing against a generalized defect in CD83Tg lymphocytes. Stimulation experiments employing spleen cells derived from CD83Tg founder 1 mice led to similar results, thus ruling out that insertion artifacts of the CD83 transgene induced the altered phenotype of CD83Tg founder 2 spleen cells (data not shown).

To allow the precise analysis of antigen-specific Ig secretion *in vitro,* we employed mice transgenic for a hen egg lysozyme-specific BCR (IgHEL) [22] that were crossed to either C57BL/6 or CD83Tg mice. Figure 3E shows that the amount of HEL-specific Ig in the serum of untreated IgHEL×CD83Tg mice was dramatically reduced compared to HEL-specific Ig in the serum of IgHEL×C57BL/6 mice. In line with this finding, *in vitro* stimulation of IgHEL×C57BL/6 and IgHEL×CD83Tg derived spleen cells clearly revealed reduced production of HEL-specific Ig by IgHEL×CD83Tg B cells compared to IgHEL×C57BL/6 B cells (figure 3F). Neutralization of IL-10 did not change the amount of HEL-specific Ig produced upon LPS activation, suggesting that the increased amount of IL-10 present in the CD83Tg spleen cell cultures was not the underlying cause of reduced Ig secretion observed in our system (data not shown).

### CD83 mediated modulation of B cell function is independent of accessory cells

**Figure 4:**
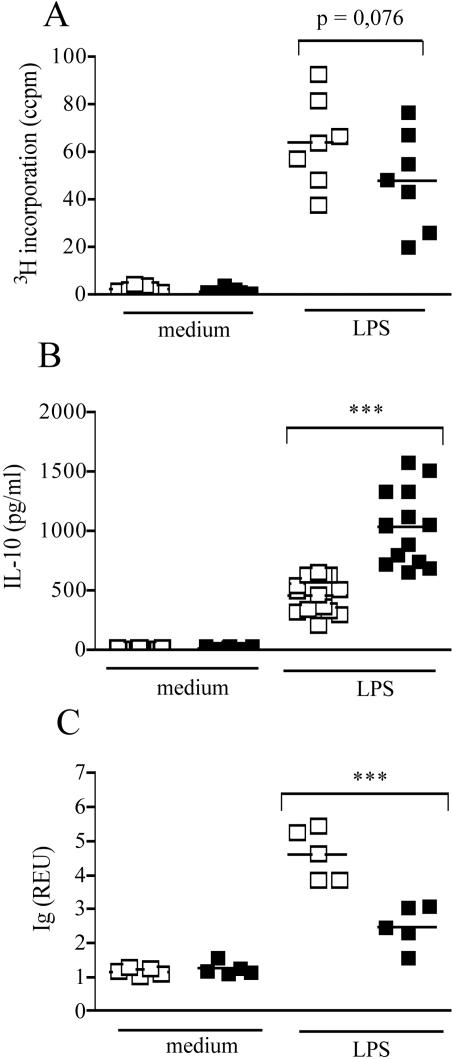
Purified CD83Tg B cells display reduced Ig and increased IL-10 response to LPS stimulation. Untouched B cells were isolated from pooled spleens of two 8–10 week old sex matched C57BL/6 (open squares) and CD83Tg (black squares) mice. 2×10^5^ B cells were cultured in 5 replicates in the presence of LPS (10 µg/ml) or no further stimulus, as indicated on the x-axis. Proliferation (4A) or IL-10 secretion (4B) were measured after 48h. 4C: Total mouse Ig in the supernatant (dilution 1∶1024) was measured after seven days by ELISA. Each dot represents an individual experiment, employing B cells purified from two pooled spleens (mean of five replicates the SEM not shown, being below 10%). The bars indicate the median and the asterisks indicate a significant difference of the median (***p<0.0005), employing students t test.

We have shown above that activated wild-type B cells rapidly upregulate CD83 and that transgenic overexpression of CD83 modulates B cell function within spleen cell cultures *in vitro*. Since CD83 is expressed under the control of the MHC-I promoter [20], it is possible that reduced Ig response and reciprocally increased IL-10 response to LPS stimulation are due to expression of CD83 by cells other than B cells. To further analyze whether the premature CD83 expression on B cells themselves modulates their own function, we stimulated purified B cells from C57BL/6 and CD83Tg mice *in vitro.* This allowed us to compare the behavior of wild-type B cells that start to upregulate CD83 three hours post stimulation to B cells that overexpress CD83 already at the beginning of stimulation. Figure 4 shows that the slightly reduced proliferation, the significantly reduced Ig secretion as well as the significantly increased IL-10 response observed in whole spleen cells (figure 3A, 3C, 3D) were also visible in purified B cell cultures (Figure 4). Stimulation of CD83Tg B cells with a combination of anti-BCR and IL-4 or with anti-BCR/anti-CD40 revealed similar results regarding proliferation while cytokine and Ig secretion were not observed at all employing these stimuli (data not shown). Reduced proliferation as well as increased IL-10 secretion in purified CD83Tg B cells were visible from day one through day five of a time course experiment thus ruling out a shift of proliferation or cytokine secretion maximum (data not shown). These results show that the increased amount of LPS-induced IL-10 was produced by the B cells themselves and that the altered function of CD83Tg splenic B cells was not dependent on CD83 expressed on accessory cells. Furthermore, these results shows that the reduced amount of Ig produced by CD83Tg spleen cell cultures does not merely reflect the slightly reduced amount of B cells within CD83Tg spleens [21]. In contrast the increase in LPS induced IL-10 secretion as well as the reduction in LPS-induced Ig secretion were even more pronounced in purified B cell cultures (figure 4B, 4C: p = 0.0001 and p<0.0006) compared to whole spleen cell cultures (figure 3C, 3D: p = 0.0032 and p = 0.018).

### *In vitro* stimulation of CD83mu spleen cells

**Figure 5:**
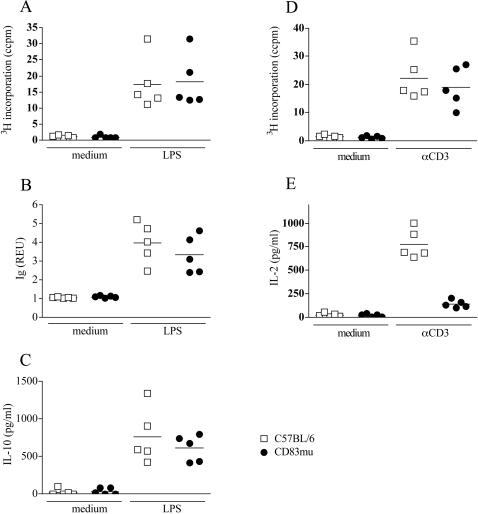
*In vitro* stimulation of CD83mu spleen cells. 2×10^5^ spleen cells derived from C57BL/6 (open squares) or CD83mu (closed circles) mice were cultured in the presence or absence of LPS (1 µg/ml) as indicated on the x-axis. 5A: Proliferation was estimated by ^3^H thymidine incorporation after 48h culture. 5B: Total mouse Ig in the supernatant was measured after seven days by ELISA and results are presented as REU of a 1:200 dilution. 5C: IL-10 in the supernatant was quantified by ELISA after 48 h. 5DE: spleen cells were cultured in the presence or absence of anti-CD3 (1 µg/ml). Proliferation (5D) and IL-2 secretion (5E) were measured after 48h culture. Each dot represents an individual experiment performed in five replicates SD being below 10%, the bars indicate the median of all five experiments performed.

Having shown that CD83 overexpression induced an increased IL-10 and reciprocally decreased Ig secretion by both CD83Tg spleen cells and purified B cells we next asked whether reduced CD83 expression would lead to an inverse phenotype. Comparison of LPS induced proliferation (figure 5A), Ig secretion (figure 5B) and IL-10 secretion (figure 5C) however, revealed no significant difference between CD83mu and wild type spleen cells despite their significantly decreased expression of MHC-II and CD86 (figure 2B, 2C). Stimulation of T cells by CD3 engagement on the other hand, induced reduced proliferation (figure 5D) and drastically reduced IL-2 secretion (figure 5E) by CD83mu spleen cells, which is in concordance with the described defective thymic maturation of CD4+ T cells in these mice [8].

**Figure 6:**
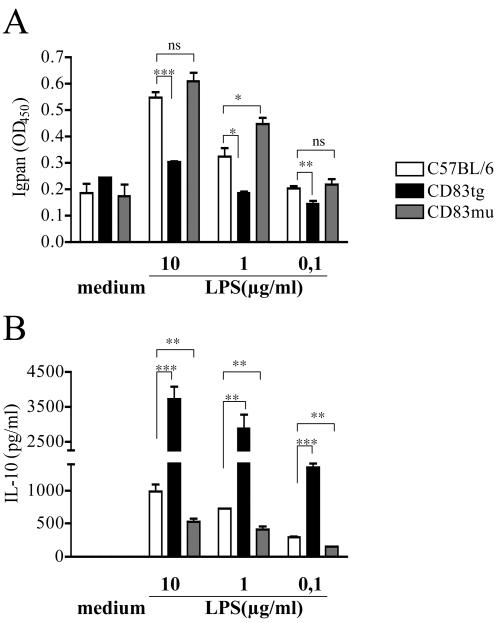
Reduced IL-10 secretion by CD83mu B cells. Untouched B cells were isolated from pooled spleens of two 8–10 week old sex matched C57BL/6 (open bars), CD83Tg (black bars) and CD83mu (dark grey bars) mice. 2×10^5^ B cells were cultured in triplicates in the presence of titrated amounts of LPS as indicated on the x-axis. 6A: Total mouse Ig in the supernatant (dilution 1∶1024) was measured after seven days by ELISA. 6B: IL-10 in the supernatant was measured after 48 h. Presented are the combined results of 3 independent experiments, error bars show SEM. Asterisks indicate a significant difference of the mean (* p<0.05; ** p<0.005 *** p<0.0005) employing students t test.

Further analysis of purified CD83mu B cells, however, revealed a slight increase in Ig secretion (figure 6A) correlated to a significantly reduced IL-10 secretion (figure 6B) by B cells derived from CD83mu mice. Although the reciprocal increase in IL-10 secretion together with the significantly reduced Ig secretion in CD83Tg derived B cells (figure 6A, 6B black bars) is more pronounced than the inverse phenotype of CD83mu B cells, this result suggests that expression of CD83 in B cells is positively correlated to IL-10 and negatively correlated to Ig secretion.

### Overexpression of CD83 on B cells interferes with calcium signaling

**Figure 7:**
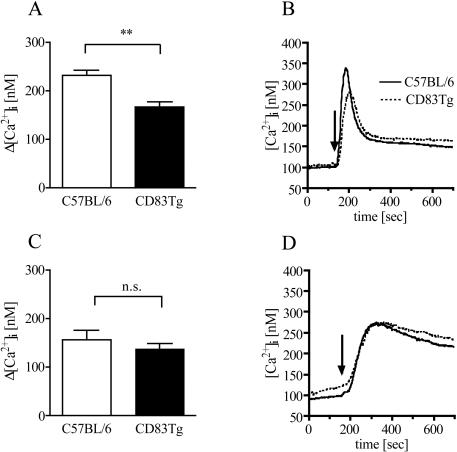
Reduced calcium signaling in CD83Tg B cells. Untouched B (7AB) or T cells (7CD) were isolated from pooled spleens of two 8–10 week old sex matched C57BL/6 (open bars) and CD83Tg (black bars) mice. Purified B and T cells were loaded with Fura-2-AM and stimulated with anti-ΒCR (Lo-MK) or anti-CD3 (145-2C11) respectively (10 µg/ml each). [Ca^2+^]_i_ was calculated from the fluorescence intensity ratio at 340 and 380 nm. 7AC: Bars represent the combined results from nine independent measurements, error bars show SEM. Asterisks indicate a significant difference of the mean. 7BD: Shown is a characteristic calcium tracing from a single experiment for B cells (7B) and T cells (7D) obtained from C57BL/6 (solid line) or CD83Tg (dotted line) mice.

While proliferation and secretion of cytokines and Ig are the final consequences of a productive B cell activation one of the earliest events is the increase in the free cytosolic calcium concentration ([Ca^2+^] _i_) within seconds after BCR ligation. Therefore, we compared the Ca^2+^ signaling in purified C57BL/6 and CD83Tg B cells and, as a specificity control, in purified T cells. Figure 7A shows that the peak Ca^2+^ signal upon BCR engagement was significantly reduced in CD83Tg B cells compared to C57BL/6 B cells (figure 7A, 7B). In contrast, TCR engagement induced comparable Ca^2+^ signaling in T cells purified from wild type and CD83Tg mice (figure 7C, 7D). These results show that transgenic overexpression of CD83 does not interfere with Ca^2+^ signaling in general, but selectively with the Ca^2+^ signaling induced by BCR engagement. The fact that the impaired Ca^2+^signaling was observed in purified B cell cultures in the absence of other CD83Tg cells again demonstrates that premature CD83 expression on B cells themselves interferes with very early events specifically in B cell activation.

## Discussion

Recently, we reported a novel function for murine CD83. Next to its well established role in the thymic maturation of T helper cells and in the regulation of peripheral T cell responses, we provided evidence that CD83 was also involved in the regulation of B cell function *in vivo* since i) CD83 was upregulated on activated B cells *in vivo*, ii) premature transgenic CD83 surface expression on the B cells themselves interfered with Ig response to TI and TD model antigen immunization and iii) CD83Tg mice displayed a dramatically reduced B cell response to *L. major* infection while mounting an unchanged protective T cell response and iv) *in vivo* application of anti-CD83 mAb induced a ten-fold increase in antigen specific IgG1 upon TI-2 model antigen immunization [21]. In the present study we pursue our findings by analyzing the role of CD83 in the modulation of B cell activation and B cell function *in vitro*.

In concordance with the inflammation-induced upregulation of CD83 demonstrated for B cells *in vivo*, CD83 was also upregulated on *in vitro* activated B cells. CD83 expression was induced with similar kinetics albeit different intensity either via TLR engagement or via BCR engagement and therefore is a consequence of both: innate and adaptive B cell activation. In line with these findings human CD83 transcription was shown to be regulated by NFκb [26], [27], a transcription factor that is induced as a consequence of either TLR or BCR signaling. We argue that the CD83 positive B cell population represents activated B cells within the spleen cell culture, since we did not detect B cells expressing the early activation marker CD69 without being also positive for CD83 within the first 12 hours of stimulation. Surface expression of CD83 on human DC was shown to be mediated by membrane translocation of the protein from intracellular stores [24] as well as by accumulation of an otherwise recirculating CD83 pool on the plasma membrane [25]. Nevertheless and despite the rapid kinetics of upregulation we did not detect significant intracellular CD83 protein stores in resting murine B cells, arguing in favor of a de novo CD83 protein synthesis in activated murine B cells. Concurring with our results CD83 upregulation by maturing human DC was shown to be also mediated by de novo protein synthesis [28].

In line with the defective humoral response and intact T cell response observed in CD83Tg mice *in vivo* [21] transgenic expression of CD83 resulted in a significant reduction of Ig secretion *in vitro* while the T cell proliferation was normal. Furthermore, as shown *in vivo*, the reduced Ig secretion of CD83Tg B cells *in vitro* was mediated by CD83 expression on the B cells themselves and not by accessory CD83Tg cells. Analyzing early signal transduction events we show that already the peak calcium signal after BCR ligation was significantly reduced in purified CD83Tg B cells. Again, this did not reflect a generalized defect in cellular calcium handling since TCR engagement induced comparable Ca^2+^ signaling in purified CD83Tg and wild-type T cells.

Strikingly, overexpression of CD83 did not result in an overall impaired function of CD83Tg B cell *in vitro* but induced a qualitative change in the phenotype of activated B cells: purified CD83Tg B cells were characterized by a significantly and reproducibly increased IL-10 production upon LPS stimulation *in vitro*. Although most of the pleiotropic functions IL-10 exhibits are anti-inflammatory [29], [30] IL-10 is regarded as a growth and differentiation factor for B cells [31], [32]. We consider it unlikely that the increased amount of IL-10 present in the CD83Tg B cell cultures caused the phenotype observed *in vitro,* since neutralization of IL-10 did not affect Ig secretion by IgHEL Tg B cells. Furthermore, the reduced calcium signaling observed in CD83Tg B cells takes place within seconds after BCR engagement i.e. well before the increased IL-10 concentration could affect B cell function. Therefore our data suggest that the increased IL-10 secretion observed in CD83Tg B cells is one feature but not the mediator of their altered phenotype. Future investigation will analyze a possible impact of increased IL-10 production by CD83Tg B cells *in vivo,* especially if CD83Tg B cells share features with IL-10 producing regulatory B cells [33]–[35] that have been described to be beneficial in various autoimmune disease models such as the recovery from EAE in resistant B6 mice [36].

Reporting a defective Ig response of CD83Tg B cells *in vivo* [21] we suggested that CD83 might represent an additional regulatory receptor such as CD22 [37] and CD72 [38] that is upregulated upon B cell activation and contributes to the regulatory mechanisms that prevent overstimulation of the B cell population [39]. The interference of premature CD83 expression with early signal transduction events upon BCR engagement as well as the decreased Ig secretion and increased IL-10 secretion of CD83Tg B cells *in vitro,* that we describe within this study, strongly support our hypothesis. The fact that CD83^−/−^ mice did not display an increased Ig response to DNP-KLH *in vivo* [7] as could be expected from mice lacking a putative negative B cell regulator does not contradict our hypothesis, since it can be explained by the reduced amount of CD4-positive T cells in these mice, as the Ig response to DNP-KLH depends on T cell help. We are currently analyzing *in vivo* B cell responses of CD83mu B cells in wild-type/CD83mu bone marrow chimeras that display a normal T cell population due to T cell maturation on wild-type thymic epithelium.

B cells derived from CD83^−/−^ mice however, were also reported to display reduced MHC-II and CD86 upregulation upon *in vitro* activation [7]. These findings apparently contradict our hypothesis, since here CD83^−/−^ B cells themselves seem to display an impaired rather than an activated phenotype. Analyzing LPS activated CD83mu B cells we, within this study, reproduced the impaired MHC-II and CD86 expression shown for CD83^−/−^ B cells. Interestingly our results clearly show that decreased activation marker expression on CD83mu B cells was not correlated to impaired B cell function since proliferation was normal and Ig secretion by LPS activated purified CD83mu B cells was even slightly increased. We also demonstrate that an increase in CD83 expression was connected to an increased expression of MHC-II and CD86 on CD83Tg B cells that again was not correlated to an increased activation state at least with respect to proliferation and Ig secretion. Finally, expression of all other activation markers and costimulatory receptors investigated was unchanged in CD83mu and CD83Tg B cells. Therefore, we argue that the altered CD86 and MHC-II surface expression observed in CD83mu and CD83Tg as well as CD83^−/−^ B cells does not reflect their activation state.

We recognize that although increased CD83 expression led to a clearly reduced Ig and increased IL-10 secretion by CD83Tg spleen cells and purified B cells, reduced CD83 expression in purified CD83mu B cells only induced a slight increase in Ig and decrease in IL-10 secretion respectively. This “weaker” phenotype may either reflect the redundancy of negative regulation in B cells [39] but could also be due to the low residual CD83 expression by the CD83mu B cells employed. Analysis of CD83^−/−^ B cells that are completely devoid of CD83 protein [7], would answer this question.

Furthermore, employing non-conditional transgenic and mutant models we cannot rule out the possibility that artificial CD83 overexpression or deficiency on pro- and pre-B cells or stromal cells during B cell maturation within the CD83Tg and CD83mu mice results in the generation of dysfunctional B cells. While the bone marrow resident pre B cell compartment appears to be normal in CD83Tg and CD83mu mice (Lüthje et al., manuscript in preparation) the increased proportion of transitional B cells within CD83Tg spleens [21] indeed suggest interference with final B cell maturation that may contribute to the phenotype. Since the maturation of B cells also depends on intact BCR signaling, this partial arrest in maturation of CD83 overexpressing B cells may again reflect a possible CD83 mediated regulation.

Taken together the combined findings presented within this manuscript that CD83Tg B cells displayed reduced Ig and increased IL-10 secretion whereas CD83mu B cells that express diminished amounts of CD83 displayed a slightly increased Ig and a decreased IL-10 production further suggests that B cell response is modulated by CD83 expression on B cells. Regarding the mechanism by which CD83 may modulate B cell function, a direct negative regulation through recruitment of Src-homology domain 2 containing proteins is not possible since CD83 lacks an ITIM motif in its predicted intracellular tail [1], [5]. Nevertheless it is conceivable that CD83 is involved in modulation of BCR signaling through association with other signaling receptors as in the CD14/TLR4 system [40]. Alternatively our data do not exclude the possibility that the interference with BCR activation is not transduced by CD83 but by a putative CD83 ligand on B cells that is engaged by overexpressed transgenic CD83. The identification of CD83 associated molecules and putative CD83 ligands will contribute to the elucidation of CD83 mediated regulation of B cell activation.
